# Reduction in the number of CGG repeats on the FMR1 gene in carriers of genetic disorders versus noncarriers

**DOI:** 10.5935/1518-0557.20170054

**Published:** 2017

**Authors:** Alexandra Peyser, Tomer Singer, Christine Mullin, Avner Hershlag

**Affiliations:** 1 Department of Obstetrics and Gynecology, Northwell Health, Division of Reproductive Endocrinology, Hofstra-Northwell School of Medicine, New York, USA

**Keywords:** FMR1, carrier screening, CGG repeats

## Abstract

**Objective:**

CGG repeat expansion on the fragile X mental retardation 1 (FMR1) gene is used to diagnose fragile X syndrome. Previous studies have discussed the correlation between the number of CGG repeats and its associated phenotypic components. The objective of this study is to determine whether the number of CGG repeats differ between carriers of genetic disorders versus noncarriers.

**Methods:**

We performed a retrospective chart review of 2867 patients who received genetic screening at our fertility clinic between June 2013 and July 2015. The number of CGG repeats on allele 1 and allele 2 on the FMR1 gene was collected and it was specified whether the patient was a carrier or a noncarrier of a specific mutation. Patients with CGG repeats greater than or equal to 45 were excluded from the study.

**Results:**

Carriers (n=759) had a reduced number of repeats compared to noncarriers (n=2024) on allele 1 (*p*=.03), allele 2 (*p*=.02) and the average of both alleles (*p*=.01). Additionally, the number of CGG repeats from the ten most carried diseases from the cohort were used and tested individually for clinical significance against the number of repeats in the noncarriers. A reduction in repeats was shown in several mutations and a few were outliers.

**Conclusion:**

Our results demonstrate that there is a significant reduction in the number of CGG repeats in carriers of genetic mutations. A larger scale study of disease carrying patients would be beneficial.

## INTRODUCTION

The fragile X syndrome is one of the most common causes of inherited mental retardation. The disorder is a result of intergenerational instability of a trinucleotide CGG repeat expansion located on the 5' translated region of the X-linked fragile X mental retardation 1 (FMR1) gene (Brown, 2002). The full mutation occurs when there are greater than 200 CGG repeats on the FMR1 gene, which results in the gene becoming fully methylated and thus silenced. The numbers of CGG repeats fall into four categories based on their stability: normal (up to 44 CGG repeats); intermediate (45-54 repeats); premutation (55-200); and full mutation (>200 repeats) (Maddalena *et al*., 2001). Some recent studies have used a lower boundary to define the beginning of the intermediate stage (e.g., 41 CGG repeats) (Hall *et al*., 2011).

Repeat expansion in the "premutation" range, between 55 and 200, can cause distinct clinical manifestations or neuropsychological changes. Disorders such as fragile X-associated tremor/ataxia syndrome (FXTAS) and fragile X-associated premature ovarian insufficiency have been associated with the premutation (Loesch & Hagerman 2012).

Many studies have correlated the number of CGG repeats with a phenotypic presentation (Mailick *et al*., 2014). For example, studies have looked at whether the number of CGG repeats differs in patients with Parkinson's Disease, Essential Tremor, and Multiple Sclerosis (Cilia *et al*., 2009; Clark *et al*., 2015; Zhang *et al*., 2009). Patients with the premutation alleles have been described to have additional phenotypic components such as developmental problems, autism spectrum disorders, attention deficit hyperactivity disorders, shyness, anxiety and seizures. Fibromyalgia and hypothyroidism have also been found to be more common in carriers of the premutation compared to controls (Rodriguez-Revenga et al.,2009; Roberts et al., 2009; Hessl et al., 2005; Bourgeois et al., 2011; Coffeyet al., 2008).

A lower-than-normal range of CGG has been associated with a myriad of clinical presentations, ranging from increased thirst and memory loss to a higher incidence of breast and uterine cancer, as well as increased risk of having a child with developmental impairment or mental disability (Mailick *et al*., 2014).

Only a few studies have examined the relationship between CGG repeats and the genotypic makeup of patients. BRCA1/2 mutation carriers have been shown to have distinctly lower CGG repeats compared to the general population (Weghofer *et al*., 2012). It has been theorized that BRCA1/2 mutations may be embryo-lethal, unless rescued by low CGG repeats in the FMR1 genes. However, this assumption has been disputed by other studies (Dagan *et al*., 2014).

The objective of this study is to determine whether the number of CGG repeats differs between carriers of various genetic disorders compared to noncarriers.

## MATERIALS AND METHODS

Genetic screening results were collected from women that came into our fertility clinic between June 2013 and July 2015. Genetic screening was done on a blood sample from the patient and it was tested for mutations in 102 clinically significant genes (Counsyl^®^, San Francisco, CA., USA). The number of CGG repeats on allele 1 and allele 2 of the FMR1 gene was also obtained from the genetic results, as well as whether the patient was a carrier or a noncarrier of one or several gene mutations. Patients with CGG repeats greater or equal to 45 were excluded from the study due to the potential of those with the ‘intermediate’ number to have Fragile X. The averages of the CGG repeats on allele 1 and allele 2 were obtained for carriers and noncarriers.

Additionally, the ten most carried diseases within our center were obtained and the CGG distribution for each disease was collected. The ten most carried diseases were: Hb Beta Chain-Related Hemoglobinopathy, Cystic Fibrosis, Pseudocholinesterase Deficiency, Spinal Muscular Atrophy, Alpha-1 Antitrypsin Deficiency, GJB2-related DFNB1 nonsyndromic hearing loss and deafness, Gaucher Disease, Familial Mediterranean Fever, Smith-Lemli-Opitz Syndrome and Achromatopsia. The average of the CGG repeats for carriers of genetic diseases was tested for clinical significance and compared to the noncarriers. The CGG distribution for each disease was also individually tested for significance against noncarriers.

Triple repeat detection was done by PCR to size the CGG repeat in the 5' UTR region of the FMR1 (NM_002024.4: c.1-131CGG[1_n]). PCR products generated from fluorescently labeled primers are detected by gel electrophoresis. Reported sizes are accurate to +/- 1 repeat for up to 200 repeats. All laboratory investigations were performed using commercial assays (Counsyl^®^, San Francisco, Ca., USA).

The distribution of the CGG repeats in women with and without a carrier mutation was compared using the statistical t-test when appropriate, at 5% significance level.

## RESULTS

In total, the study included 2,867 women who received genetic screening. Of those, 843 (mean age 35.9±5.4y) were carriers of one or more genetic diseases, and 2024 (mean age 35.7±5.0y) were noncarriers. 84 women were excluded from the carrier cohort because they had CGG repeats greater than or equal to 45. Therefore, 759 carriers of a genetic mutation were included in the study.

Table 1 outlines the average number of CGG repeats for carriers, noncarriers and the top ten carrier mutations at our center. Carriers had lower CGG repeat values compared to noncarriers for allele 1 (*p*=.03), allele 2 (*p*=.02) and the average of both alleles (*p*=.01). Allele 1 repeats in carriers of Alpha-1 Antitrypsin, Gaucher, Familial Mediterranean Fever and Smith-Lemli-Opitz Syndrome had significantly lower repeats than noncarriers. (*p*=.01, *p*=.02, *p*=.02, *p*=.01, respectively). Allele 2 repeats in carriers of Alpha-1 Antitrypsin, Gaucher and Smith-Lemli-Opitz Syndrome had lower repeats than noncarriers (*p*=.02, *p*=.01, *p*=.05, respectively). The average of both allele 1 and allele 2 in Alpha-1 Antitrypsin, Gaucher and Smith-Lemli-Opitz Syndrome carriers demonstrated significantly reduced CGG repeats than noncarriers. (*p*=.003, *p*=.004, *p*=.009). No significant difference was noted in any of the other CGG distributions within the other diseases.

**Table 1:** Average number of CGG repeats for carriers and noncarriers on allele 1, allele 2 and the average of alleles 1 and 2.

Disease	CGG Allele 1 (Mean)	CGG Allele 2 (Mean)	Avg. of Both Alleles
Hb Beta *(n=93)*	28.20±3.7	31.58±4.0	29.90±3.3
Cystic Fibrosis *(n=90)*	27.92±3.6	31.39±3.6	29.66±3.0
Pseudocholinesterase *(n=65)*	27.62±4.2	31.97±4.2	29.79±3.3
SMA *(n=48)*	28.23±3.7	31.06±2.9	29.65±2.4
GJB2 *(n=47)*	27.60±4.2	31.21±4.7	29.40±3.7
Gaucher *(n=43)*	26.51+ 4.7	30.33±3.3	28.42±3.4
Alpha-1 Antitrypsin *(n=39)*	26.41±4.9	30.41±3.3	28.41±3.5
Smith-Lemli-Opitz Syndrome *(n=35)*	26.43±4.3	30.60±2.3	28.51±2.7
FMF *(n=34)*	26.41±4.8	31.74±4.1	29.10±3.6
Achromatopsia *(n=30)*	28.87±3.4	31.00±2.9	29.93±2.3
Noncarriers *(n=2024)*	27.82±3.9	31.55±3.5	29.69±3.3
Carriers *(n=759)*	27.50±4.3	31.25±3.6	29.37±3.2

Abbreviations: Hb-Beta, Hb Beta Chain-Related Hemoglobinopathy;

SMA, Spinal Muscular Atrophy;

GJB2, GJB2-related DFNB1 nonsyndromic hearing loss and deafness;

FMF, Familial Mediterranean Fever.

## DISCUSSION

This study evaluated whether there is a correlation between the numbers of CGG repeats in carriers of genetic mutations versus noncarriers, to determine whether CGG repeats differ between the two groups. We evaluated 2867 women who were screened for genetic mutations. Overall, carriers had a lower number of CGG repeats than noncarriers. However, several mutations were outliers. Weghofer *et al*. (2012) described an association between low CGG repeats and BRCA1/2 positive women. They suggested that low numbers of CGG’s rescued embryos carrying the BRCA1/2 mutation. If a human embryo carries a low allele (<26), then the embryo is able to overcome the BRCA1/2- associated embryo lethality. Additionally, they hypothesized that BRCA1/2 mutations may somehow be able to influence CGG repeat expansion.

Another trinucleotide pattern, the CAG repeat, has been found to be associated with a higher risk of cryptorchidism in patients with shorter repeats. It has been hypothesized that there is an indirect influence whereby the shorter CAG repeats create lower testosterone levels and, therefore, influence the androgen receptor mediated genomic pathway associated with cryptorchidism (Davis-Dao *et al*., 2012).

The mechanism by which the carrier status can cause a reduction in the number of CGG repeats or vice-versa could be a result of an indirect relationship between the transcription of the FMR1 gene and the genes responsible for the carrier mutation. Mechanistic investigations are needed to identify these indirect or direct effects.

A limitation to our study is the small sample size. Indeed, a larger cohort with more carriers would be beneficial.

In conclusion, this preliminary investigation suggests that carriers of genetic mutations have less CGG repeats on the FMR1 gene when compared to noncarriers. Larger epidemiological studies of disease carrying patients as well as analysis of genomic pathway mechanisms may be beneficial.

## HUMAN STUDIES AND INFORMED CONSENT STATEMENT

All procedures followed were in accordance with the ethical standards of the responsible committee on human experimentation (institutional and national) and with the Helsinki Declaration of 1975, as revised in 2000. Informed consent was obtained from all patients for being included in the study. The Northwell Health Institutional Review Board reviewed and approved this study.
